# A Christmas "Caution."

**Published:** 1912-12-21

**Authors:** Ellis Marsland

**Affiliations:** Gen. Hon. Secretary.


*To the Editor of* The Hospital.

### A Christmas "Caution."

*To the Editor of* The Hospital.

Sir,—We would ask you very kindly to co-operate in our endeavours to caution all concerned regarding the fire dangers to be met with in connection with the Christmas festivities and at Christmas parties, as fires on these occasions frequently result in personal injury, and often even fatalities. We enclose a copy of the Committee's official "Caution," and suggest your kindly publishing it in the public interest.—We are, Sir, yours very truly,

For the British Fire Prevention Committee,

Ellis Marsland, Gen. Hon. Secretary.

The "Caution" referred to reads as follows :—

Xmas Trees : Xmas trees should not be placed near window curtains or in positions in which a draught may cause inflammable draperies, etc., to be blown on to the lights. Evergreens : All dry shrubs are easily ignited, especially the fir-trees used at Xmas, as they contain a considerable quantity of resin. Candles : Candles are easily bent out of shape, even by the slight heat of another light below. Bent candles drop down and set things alight. Paper Lanterns : All paper and similar lanterns should be hung by wire, and during the period they are lighted should be watched to see they do not swing. A swinging "Chinese lantern" easily catches fire. Tissue Paper : Tissue paper (unless properly treated) should not be used as a decoration or covering for illuminated globes. Cotton Wool : Cotton wool (unless properly treated) should not be used to represent snow, as it is highly inflammable. Asbestos fibre or slag wool are good substitutes. Celluloid : Celluloid, being a highly inflammable material, should not be used on Xmas trees or in decorative schemes. Matches : Do not leave matches within the reach of children. Children should not be allowed to light Xmas tree or other candles unless adults are present. They frequently set fire to their clothing instead. Electric Wiring : Do not make the slightest change in electric wiring without consulting a competent electrician or an electrical inspector.

"Snap Dragon" : In playing "Snap Dragon," the players should be warned not to shake any lighted spirit upon muslin, flannelette, or other highly inflammable clothing. Players should remove any such accessories as celluloid bangles, strings of celluloid "beads," or hair ornaments.

